# How B-Cell Receptor Repertoire Sequencing Can Be Enriched with Structural Antibody Data

**DOI:** 10.3389/fimmu.2017.01753

**Published:** 2017-12-08

**Authors:** Aleksandr Kovaltsuk, Konrad Krawczyk, Jacob D. Galson, Dominic F. Kelly, Charlotte M. Deane, Johannes Trück

**Affiliations:** ^1^Department of Statistics, University of Oxford, Oxford, United Kingdom; ^2^Division of Immunology and the Children’s Research Center, University Children’s Hospital, University of Zürich, Zürich, Switzerland; ^3^Oxford Vaccine Group, Department of Paediatrics, University of Oxford and the NIHR Oxford Biomedical Research Center, Oxford, United Kingdom

**Keywords:** Ig-seq, antibody modeling, B cell, Antibodies, Developability, computational modeling, Next-generation sequencing

## Abstract

Next-generation sequencing of immunoglobulin gene repertoires (Ig-seq) allows the investigation of large-scale antibody dynamics at a sequence level. However, structural information, a crucial descriptor of antibody binding capability, is not collected in Ig-seq protocols. Developing systematic relationships between the antibody sequence information gathered from Ig-seq and low-throughput techniques such as X-ray crystallography could radically improve our understanding of antibodies. The mapping of Ig-seq datasets to known antibody structures can indicate structurally, and perhaps functionally, uncharted areas. Furthermore, contrasting naïve and antigenically challenged datasets using structural antibody descriptors should provide insights into antibody maturation. As the number of antibody structures steadily increases and more and more Ig-seq datasets become available, the opportunities that arise from combining the two types of information increase as well. Here, we review how these data types enrich one another and show potential for advancing our knowledge of the immune system and improving antibody engineering.

## Introduction

**Figure 1:**
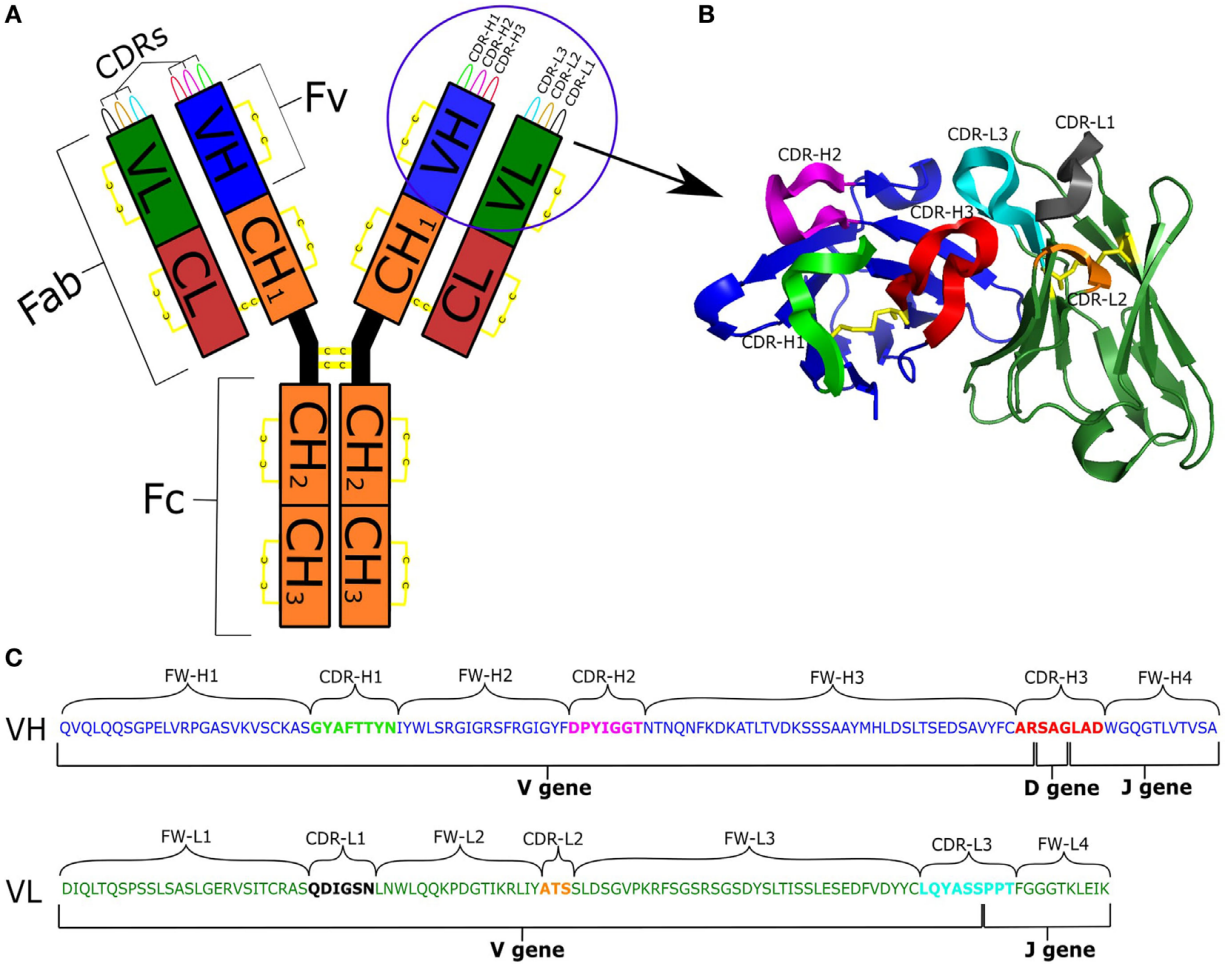
**(A)** Schematic representation of an antibody IgG structure. **(B)** Structure of the Fv region. **(C)** Genetic composition of VH and VL chains [IMGT numbering (9)]: VH is colored blue; VL is green; CDRs are labeled and depicted in different colors; and disulfide bonds are in yellow.

Antibodies are proteins produced by the B cells of jawed vertebrates. Their primary function is to recognize structural sequence motifs (epitopes) within molecules (antigens) usually related to pathogens, which may lead to direct neutralization of those pathogens or their toxins. Further functions of antibodies are activation of the complement system or tagging of antigens for elimination by other immune pathways. Antibodies have the capacity for binding an extraordinary variety of epitopes as a result of their sequence diversity, which is estimated at 10^13^ unique molecules in the human antibody repertoire (1). An antibody is a large complex molecule (~150 kDa). It can be divided into two parts, the crystallizable fragment (Fc) and the antigen binding fragment (Fab). The Fab fragment is further split into constant and variable regions. There are five possible main Fc portions in humans, and which one is used on a particular antibody is governed by the process of class switching (2). The variable region (Fv) is composed of two domains called the heavy (VH) and light (VL) chains. Within each B cell, the antibody Fv domains are built by somatic recombination between V(D)J segments (3, 4). Upon antigen recognition, somatic hypermutation introduces further diversification into the naïve Fv domains (5). Within each of the VL and VH chains lie three hypervariable loops, the complementarity determining regions (CDRs), which are the most diverse parts of the antibody (Figure 1). These loops form the majority of chemical interactions with antigens, thus defining the antigen-binding region, the paratope (6). The CDR3 of the heavy chain (CDR-H3) is the most diverse of the CDRs as it is being formed at the join between the V, D, and J gene segments and subject to high levels of hypermutation. As a result of this diversity, CDR-H3 plays a key role in antigen recognition and binding (7). The non-CDR sections of the variable domain are called the framework. Framework positions next to CDRs along with CDR sequence govern the structural shape of the loops (8).

The properties of antibodies, in particular designable antigen recognition specificity and binding affinity, have made them useful as diagnostics and research agents as well as the most successful class of biopharmaceuticals (10). Although small molecules constitute the largest proportion of potential therapeutics in clinical trials, the antibody market is steadily growing, with new antibody approvals at a rate of about four per year. As of 2016, five out of the 10 best-selling drugs worldwide were recombinant monoclonal antibodies (11).

Successful exploitation of antibodies relies on our ability to interrogate their diversity and function. Application of next-generation sequencing of immunoglobulin gene repertoire (Ig-seq) to antibody profiling is able to produce comprehensive snapshots of the repertoire diversity (12). However, most Ig-seq techniques are currently unable to perform sequencing of paired heavy–light antibody sequences or to obtain an immunoglobulin gene repertoire solely from antibody-secreting B cells (13–15). Advances in liquid chromatography tandem-mass spectroscopy (LC-MS/MS) now allow high-throughput analysis of serum antibodies at the amino-acid sequence level (16, 17). Previously transcriptomics and Ig-seq datasets have been used to deconvolute MS spectra of serum antibodies into constituent full-length entities (18). Such combined Ig-seq and LC-MS/MS techniques have provided new insights in vaccination and autoimmunity studies (19, 20). Recent advances in computational tools that integrate *de novo* antibody sequencing, error correction data, and sequence homology databases now permit an accurate assembly of full-length antibodies based on the remit of LC-MS/MS spectra alone (21).

The biggest advantage of Ig-seq and LC-MS/MS techniques is their high-throughput nature. This means that the methods provide a broad-brush description and quantification of antibodies in the repertoire. However, this will often include inaccurate data caused by PCR or sequencing errors. The limitation of Ig-seq and LC-MS/MS methods is that they provide sequence information only, whereas it is the shape/structure of an antibody that determines its exact biological function. For instance, antibody CDRs with low-sequence identities can adopt structurally close shapes, and hence present conformationally similar, though perhaps chemically different, binding sites (22). Knowledge of antibody structure is vital for inferring chemistry of antigen recognition as well as allowing binding site comparison between antibodies. Current experimental determination of antibody structures is achieved by X-ray crystallography or NMR spectroscopy. However, collecting such detailed experimental information limits the rate of analysis to the level of individual or a small number of antibodies (23).

To help tackle the rising costs and time required for engineering and characterization of antibodies, a number of computational tools have been developed that can facilitate experimental efforts. Computational methods are used to profile the physico-chemical properties of antibodies, predict antibody–antigen contacts, and redesign antibody–antigen complexes (24, 25). The tools can be broadly divided into those that require only the sequence of an antibody as input and those that require the structure of the antibody. The inclusion of structural information where available has been shown to improve prediction of most properties over sequence-based methods (26). These improved predictions are only possible if a native structure or an accurate model of the antibody is available.

Since the structure of an antibody is key to its function and high-throughput crystallographic determination of the structures of every antibody is currently not feasible, computational modeling techniques may aid to reduce attrition in the biopharmaceutical industry and to accelerate drug discovery (27). The development of systematic relationships between the antibody information gathered from Ig-seq and techniques such as X-ray crystallography, NMR spectroscopy, and tandem LC-MS/MS could radically improve our understanding of antibody biology. As the number of antibody structures steadily increases and more Ig-seq datasets become available, the opportunities that arise from combining them increase as well. As of October 9, 2017, more than 2,860 antibody structures were available in the Protein Data Bank (PDB) (28) as identified by the Structural Antibody Database (29). The publically available volume of sequences produced from Ig-seq experiments is now in the hundreds of millions (30). In this manuscript, we consider the information obtained from high-throughput sequencing experiments and antibody structures. We review how these datasets can enrich one another and with the help of computational techniques, advance our knowledge of antibody diversity, maturation, and selection and pave the way for improved antibody engineering.

## Immunoglobulin Gene Repertoire Sequencing Technologies

Ig-seq offers high-throughput characterization of immunoglobulin gene sequences at great depth and typically includes several B-cell samples in a single-sequencing run. By controlling the number of samples that are combined and the number of B cells contained therein, it is possible to obtain a large fraction of an immunoglobulin repertoire from a sample. The potential applications of Ig-seq include vaccine and drug development as well as immunodiagnostics (12, 31, 32). Such applications rely on our ability to efficiently identify the population of antibodies responding to an antigen challenge. Ig-seq has already been successfully applied to isolate antigen-specific antibodies from immunized animals in conjunction with common laboratory screening platforms such as phage display (33) or hybridoma (34) or even when the screening step was omitted (35). Furthermore, amino-acid sequence convergences in the CDR-H3 have been observed in the response to a variety of antigens, and may serve as an additional way to isolate antigen-specific antibodies through identifying sequences common among several individuals exposed to the same antigen (30, 36–39).

Heavy and light chains are products of two independent mRNA transcripts that co-assemble into full-length immunoglobulin molecules in the endoplasmic reticulum of the B cell. However, cognate pairing is lost after B-cell bulk lysis prior to Ig-seq and most Ig-seq studies therefore only consider heavy chains (12). However, for human and mouse native pairing is crucial for antibody folding, stability, expression, and antigen binding (40–42). Furthermore, information on the heavy/light chain dimer is required to create an accurate three-dimensional (3D) model of the Fv region and of its antigen-binding pocket which is essential for rational antibody engineering (43). Such models can map antibody sequences to structural space (44), identify the paratope and its physico-chemical properties (45), interrogate the mode of interaction with antigens (46), and predict antibody developability properties (47, 48). Predicting or experimentally obtaining the native VH/VL pairing of the antibody is therefore crucial for our understanding of antibody biology and our ability to engineer these molecules (49).

Several approaches have been devised to circumvent the loss of native pairing in current Ig-seq experiments. Reddy et al. (35) assigned VH/VL pairs based on relative variable chain frequencies in VH and VL chain Ig-seq datasets. This methodology required an accompanying VL Ig-seq dataset and does not always produce antibodies with good pharmacodynamics properties, indicating that it is not always accurate (35). Researchers have also used protein expression platforms, such as recombinant cell lines or phage display, to assign VL to VH chains in a combinatorial fashion followed by experimental screening to identify productive VH/VL combinations (20, 50). Dekosky et al. (15, 51) published the first high-throughput paired VH/VL gene sequencing approach by using single-cell linkage PCR to physically join the VH and VL chains prior to Illumina sequencing. The limitation of this approach is that the current Illumina read length cannot cover the entire paired sequence, so the analysis is restricted to only CDR-H3, CDR-L3, and neighboring framework 4 and proximal positions of framework 3 of respective chains. Once sufficient paired datasets are available, these can potentially act as a reference for guiding computational pairing when VH-only Ig-seq is performed (52). Paired Ig-seq techniques currently yield smaller dataset sizes than unpaired sequencing––for instance, there were 200k sequences for the paired dataset from Dekosky et al. (15) as opposed to 40-m unpaired VH sequences in a recent study (53). The unprecedented speed and depth of Ig-seq techniques both paired and unpaired is unfortunately accompanied by high-sequencing error rates as discussed below.

The four main high-throughput sequencing platforms used to interrogate the immunoglobulin gene repertoire are Illumina, Roche 454, PacBio, and IonTorrent (39, 54–57). Earlier studies often used the Roche 454 technology as it offered greater read lengths than the Illumina methodology. In recent years, Illumina sequencing platforms are usually preferred as they have increasing read length, higher read depth, lower error rates, and lower costs per base (57, 58). Employment of unique molecular identifiers (UIDs) now permits sequencing of the entire antibody chain together with a fragment of a constant domain which holds antibody isotype information (59, 60). Unfortunately, any high-throughput Ig-seq technique suffers from significant error rates (61). Sequencing error can be introduced into Ig-seq datasets from incorrect base calling and sequencing primer artifacts, and has distinct features depending on the sequencing platform used. Error and biases can also originate from the process of preparing sequencing material including reverse transcriptase and polymerase error, amplification of nonproductive V(D)J variable domains during DNA sequencing and multiplex PCR amplification biases (62, 63). Such error may result in the overestimation of the actual number of unique clones in an Ig-seq dataset (62).

Several computational and experimental approaches have been developed to identify and remove or correct erroneous reads (58, 63), though no single-error correction strategy is currently widely used in Ig-seq repertoire analysis (30, 58). In particular, the recent application of UID to Ig-seq can help to correct errors in sequenced transcripts by generating a consensus of reads originating from the same mRNA molecule. As many studies are confined to CDR-H3 analysis, erroneous reads may also be corrected for by using a consensus CDR-H3 sequence for analysis following CDR-H3 clustering (39, 51, 64).

## Antibody Structural Properties

The structure of an antibody is crucial in order to understand its function. Antibody–antigen recognition relies on the 3D conformation of the antibody binding site, the paratope, in relation to the cognate epitope on the antigen. In their 3D form, antibodies adopt a Y-shape conformation which can exist in monomer (IgG, IgD, and IgE), dimer (IgA) or pentamer (IgM) forms in humans (65). Several disulfide bonds help to maintain the immunoglobulin fold (Figure 1). One set of disulfide bonds hold the heavy constant domains together in the hinge region and another set connects the light and heavy chains (66). Intra-variable domain cysteine pairs play a crucial part in shaping the antibody Fv region and artificial disruption of these bonds leads to impaired stability, folding and antigen recognition (67). These cysteines therefore have a crucial role in delineating the structural features of an antibody.

Equivalent residue positions across immunoglobulin sequences and structures can be identified by applying an antibody numbering scheme. Several numbering schemes have been developed to confer consistency and standardization on antibody sequence annotation (9, 22, 68–71). The most commonly used scheme in Ig-seq analysis is the IMGT scheme (12, 39). This numbering was built considering both structural and sequence information (9). The IMGT scheme supports symmetrical amino-acid insertions inside CDRs which ensures that structurally equivalent resides will be annotated the same regardless of CDR length. In contrast, Chothia numbering is often used by structural biologists for its simple CDR loop indel management and inherently structural focus (69, 71).

One of the principal differences between numbering schemes is how they define CDRs. Wu and Kabat (68) were the first to discover and define CDRs as portions of Fv chains that display high-sequence entropy, but as with numbering schemes, there is not a single widely adopted CDR definition and different schemes are used for legacy reasons or for specific features (such as insertion management in IMGT). The different numbering schemes define antibody CDR positions very consistently with the exception of CDR-H1 and CDR-H2 (70). Structural analysis of CDR loops has suggested that all CDRs, except for CDR-H3, adopt a restricted number of conformations, termed canonical classes (22, 72). The canonical classes link sequence patterns to a defined structure (22, 44). This enables the prediction of canonical class structure from sequence. Over the last 30 years, there have been several attempts to cluster CDR sequences/structures (22, 44, 69, 70, 72, 73). On the sequence level, the presence of certain cluster defining key residues indicates the shape the loop can adopt (22, 69, 73). Hence, some changes to the canonical CDRs can be tolerated with no explicit change to loop conformations. The different clustering methods tend to recapitulate previously found groups and find new canonical forms as a result of new data. Most algorithms incorporate CDR loops into clusters with the same number of residues (note that the number of residues varies with different CDR definitions). More recently, Nowak et al. (44) created a novel method of defining length-independent canonical classes based on findings that loops of mismatching lengths can be structurally related. This method allowed fast and accurate structural assignment of a far wider spectrum of canonical CDRs from Ig-seq datasets into fewer canonical clusters (44).

**Figure 2:**
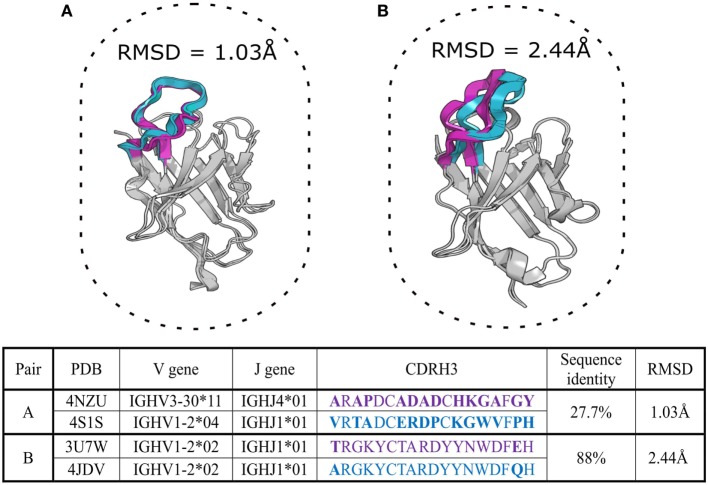
Two aligned pairs of VH chains extracted from SAbDab, the antibody structural database (29). Complementarity determining region-3 of the heavy chain (CDR-H3) sequences in pair **(A)** belong to different CDR-H3 clonotypes but adopt very similar structural configurations with a root mean square deviation (RMSD) of ~1 Å. Pair **(B)** includes germline precursor (4JDV) and matured (3U7W) anti-gp120 antibodies (78, 79). Although CDR-H3 sequences of pair **(B)** are members of the same clonotype, the RMSD shows that their CDR-H3 shapes are structurally distinct (RMSD > 2 Å). CDR-H3 loops and their amino-acid sequences are in purple and cyan colors, mismatched amino acid are in bold. The RMSD of the backbone atom positions of proteins provides a pairwise measurement of the three-dimensional dissimilarity between two sets of coordinates where solved or predicted structures are available. Sub-Angstrom RMSD indicates structurally identical shapes, while an RMSD value greater than 2 Å for a short segment indicates structurally distinct configurations (80).

Complementarity determining region-3 of the heavy chain shows a high degree of sequence, length, and structure variation. Due to this diversity, it has so far proved impossible to classify CDR-H3 loops into canonical classes in the manner of the other CDRs. It has been proposed that CDR-H3 can be categorized into “bulged” or “extended” conformations based on the presence of asparagine at position 116 (IMGT numbering) (74, 75). However, increasing knowledge of CDR-H3 structural diversity has shown that the CDR-H3 bulged/extended configuration is difficult to predict solely from sequence (76). The relationship between sequence and structure in CDR-H3 can be important in Ig-seq as current approaches of clonotype assignment are based on CDR-H3 similarity. In this review, we define clonotypes by the presence of identical V, J genes, matching CDR-H3 lengths and CDR-H3 sequence identities greater than 85% (77). However, structural data show that CDR-H3 sequences within distinct clonotypes (sequence-dissimilar) can adopt similar 3D conformations, while those in the same clonotype (similar sequences) can adopt different 3D conformations (Figure 2). This suggests that the sequence alone is not a reliable indicator of similarity/difference between structures and therefore cannot reliably indicate similar/different binding sites, functional properties and clonotype assignment.

The discrepancy between traditional clonotype assignments and native structure only illustrates how 3D information could be used to draw much more meaningful comparisons between antibodies in an Ig-seq dataset. Such comparisons should not be confined to CDR-H3 alone, but can be extended to the canonical CDRs and the entire Fv region, allowing for much more accurate grouping of functionally related antibodies.

## Computational Tools Leveraging Antibody Structure Information

The increasing number of potential applications of antibodies as therapeutics has led to the development of computational tools which aim to streamline discovery pipelines. Some groups have already demonstrated the viability of *in silico* antibody engineering methodologies in conjunction with experimental workflows (81–84). Computational methods can be broadly divided into those that require a sequence as input and those that require a structure. Methods that require a structure as input accept experimental as well as computational models of the antibody. The large number of experimentally determined antibody structures has enabled researchers to rapidly and accurately model antibodies by leveraging homology methods (8, 85). Below we review current antibody modeling approaches and their applications.

### Computational Antibody Modeling

**Figure 3:**
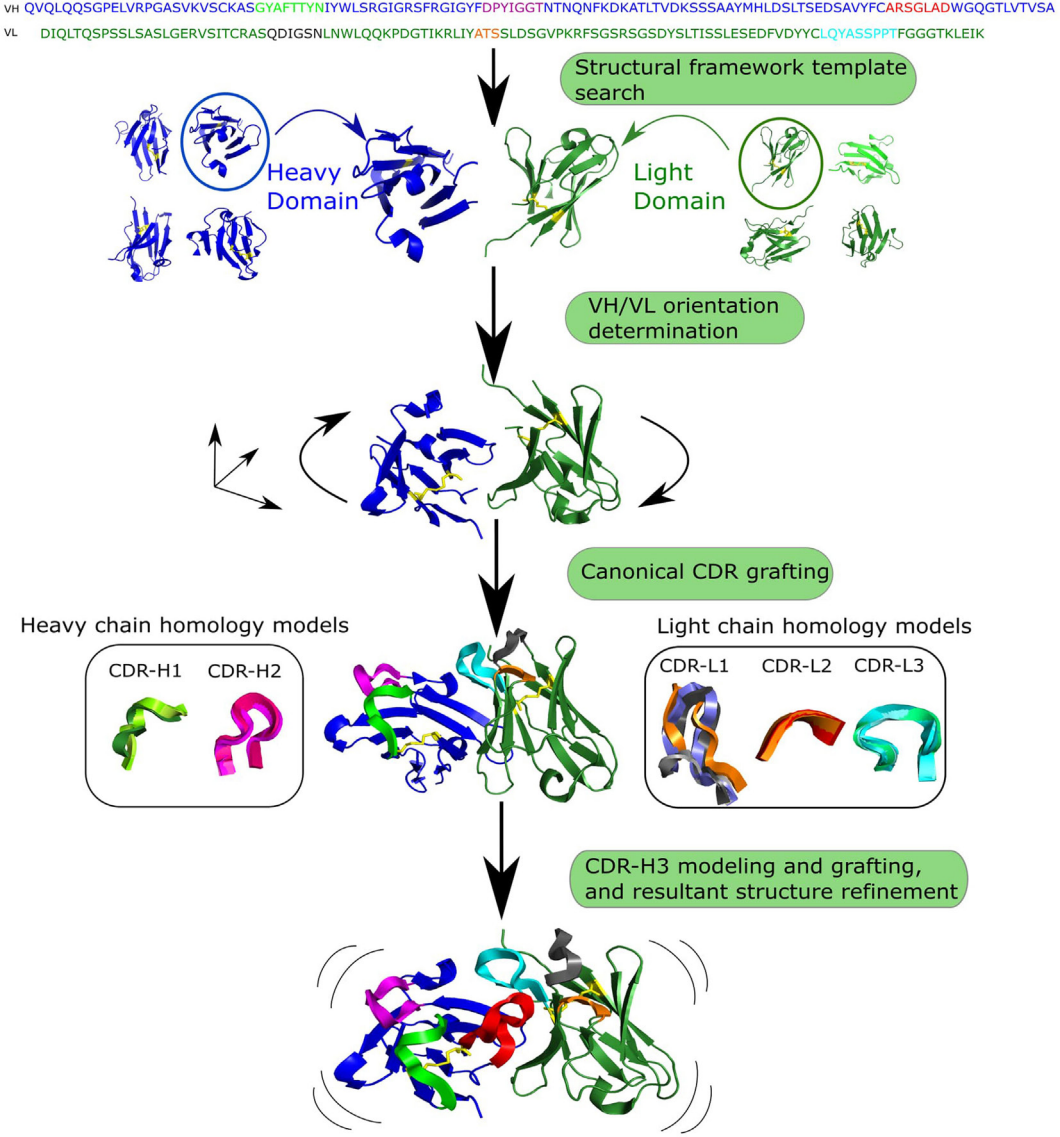
Generalized workflow of antibody modeling. First, heavy and light chain frameworks are determined by homology modeling using templates from known structures. Next, the VH/VL orientation is calculated. The third step is modeling non-H3 complementarity determining regions (CDRs), followed by modeling and grafting of CDR-H3 onto the pre-assembled scaffold. Finally, sidechains are added to the resultant structure and it is refined.

The standard antibody modeling workflow includes four steps (Figure 3) (8, 86, 87). The first step is homology modeling of the VH and VL frameworks. The framework template can either be selected by sequence identity to the full-length chain (87) or to individual framework regions (8). Due to framework structure and sequence invariance, current computational tools can model framework structures very accurately (sub-Angstrom precision) (80). The second step is determining the VH/VL orientation, which can be achieved by copying the orientation angle from structures with high Fv sequence identity using VH/VL orientation methods such as AbAngle (88), analytical estimation of the angle using energy functions (89), tailored protein–protein docking (49) or structure-trained machine learning (90). Once the VH/VL orientation is set, it constrains the geometry of the binding site, allowing for the third step, which is modeling of non-H3 CDRs. At this stage, either the canonical classes are used (91) or template-based modeling such as FREAD (92) or ABGEN (93). In the final step, CDR-H3 is modeled using either homology or *ab initio* techniques (94). The resultant antibody model is refined for feasibility of dihedral angles from Ramachandran distribution, side chain orientations and side-chain clashes (89).

Homology modeling approaches can be fast at generating models if a template structure is available. Models can be created using online services: PIGSpro (86), Kotai Antibody Builder (95), and ABodyBuilder (8). Homology modeling is highly dependent on the availability of a similar template structure in current databases, which can be a problem for CDR-H3 where templates for longer loop length are often unavailable (94). This lack of templates is primarily due to the huge diversity of CDR-H3 shapes (96). An alternative to homology methods in such cases is *ab initio* modeling which does not rely on knowledge of already solved structures. These modeling methods create a large number of potential conformations, often referred to as decoys (97), which makes them computationally expensive compared with homology methods. *Ab initio* approaches include RosettaAntibody (98) and PLOP (99). RosettaAntibody is accessible online *via* the ROSIE (100) website, where a quick antibody modeling option is available which omits the step of intensive searching for low-energy CDR-H3 conformations. Hybrid loop modeling methodologies leverage the advantages of both modeling paradigms. For instance, Accelrys creates an initial loop model with a knowledge-based approach followed by *ab initio* loop refinement (101). More recently, a novel CDR-H3 modeling tool, Sphinx, was developed (102), inspired by the length-independent canonical CDR clustering of Nowak et al. (44). Sphinx outperformed all modeling tools on CDR-H3 structure prediction in an *ex post facto* comparison to the antibody modeling assessment (80). Despite development of different approaches, no single tool currently exists that is able to reliably model native CDR-H3 configurations. Accurate predictions of the CDR-H3 specifically and other CDRs in general are crucial to structurally characterize the antibody–antigen complex.

Performance of antibody modeling tools has been assessed in two blind studies, AMA-I and AMA-II (80, 103), where several computational tools were benchmarked against a small number of X-ray solved but unpublished antibody crystal structures. Models of frameworks and canonical CDRs are usually accurate within 1–1.5 Å root mean square deviation (RMSD), respectively (see Figure 2 for description of RMSD), which is very close to native structure. However, CDR-H3 prediction remains the biggest hurdle for computational antibody modeling as average accuracies for this step ranged between 2 and 3 Å RMSD, indicating a decidedly different structure to the native fold. Predictions of this quality are usually not suitable for rational design applications (80, 104).

AMA-II suggested that antibody modeling tools on average produce models of approximately similar accuracies with higher RMSD for longer loop lengths. However, the time required is radically different between homology and *ab initio* approaches (80). Homology modeling can produce a model on average in under a minute [ABodyBuilder (8)], whereas *ab initio* approaches may require up to tens of CPU hours per model [RosettaAntibody takes 482 CPU hours on average per model (100)]. To be able to use a fast homology method a suitable template is needed. Such templates are becoming more frequently available as the number of solved antibody structures increases (29). In order to model millions of sequences in a typical Ig-seq dataset, speed is crucial. Modeling at such high throughput can currently only be achieved by tools such as ABodyBuilder, which is able to generate a model within ~30 s (8). However, further increasing the rate and accuracy of antibody modeling, and developing new ways of speeding up CDR-H3 prediction, are needed if we are to structurally characterize complete Ig-seq datasets.

The accuracy and speed of some computational tools mean that thousands of sequences from Ig-seq datasets can be modeled. Such structurally annotated Ig-seq datasets allow more relevant comparisons of CDRs, binding sites and thus a more accurate grouping of molecules (Figure 2). The improved capacity to compare and group antibodies allows us to better visualize the antibody structure space and to investigate structural convergences of paratopes, which can be important for vaccine development (36, 37). In addition, modeled Ig-seq data can be used as input for several computational tools which annotate structure-derived antibody properties, such as therapeutic viability of the molecule (105).

### Computational Prediction of Developability

Developing an antibody of high specificity and affinity against a target is only the initial step in engineering a therapeutic molecule. The resulting antibody can carry an array of risks, collectively described as developability, which includes low-expression yields, high-aggregation propensity, and off-target effects (106, 107). In the process of identifying therapeutic candidates, structurally mapped Ig-seq data can be computationally further refined for entities that pass developability criteria (45).

High-aggregation propensity is one of the most undesirable features of antibody therapeutics. Since aggregation is related to the hydrophobicity of the molecule, knowledge of structure is crucial as it allows the calculation of solvent accessible surface area. Structure-based aggregation propensity prediction tools operate by either locating surface-exposed aggregation hot spots and/or leveraging physico-chemical properties of the structure (105, 108). AGGRESCAN3D, a tool inspired by identification of hot spots in the beta amyloid peptide, distinguishes between buried, conformation engaged, and solvent-exposed aggregation prone hydrophobic patches (48). The drawback of this method was that it was not initially designed for antibodies. The Developability Index (DI) was designed for antibodies and is a structure based computational tool that quantitatively assess antibody’s propensity to aggregate (105). The DI function considers the net charge of the full-length antibody and hydrophobicity of solvent-exposed sidechains of CDRs.

Such computational tools can be employed early in drug development pipelines to either isolate therapeutically viable drug candidates from the entirety of Ig-seq-derived antibody repertoire (47). Application of such structurally oriented tools requires large-scale modeling of Ig-seq datasets. Nevertheless, to date, there have not been many attempts to combine Ig-seq with structural and computational methods systematically.

## Combining Ig-seq, Structural, and Computational Approaches

Current approaches to delineate immune repertoires usually employ Ig-seq methodology only, remaining firmly within the remit of information that can be derived from sequences (31, 109, 110). The only study which has attempted to combine paired Ig-seq and structural information to characterize antibody 3D space was that of Dekosky et al. (45). Using high-throughput RosettaAntibody modeling, more than 2,000 models in naïve and antigen-experienced Ig-seq datasets were analyzed. These models helped to obtain a set of structural descriptors such as net charge, surface hydrophobicity of solvent accessible surface area for computationally determined paratopes. However, the choice of methodologies for this study imposed several limitations. Paired VH/VL data did not contain information about the full-length Fv region. Hence, all paired reads had to be completed using respective V germline gene sequences. Moreover, RosettaAntibody modeling speed only permitted the prediction of structure of 1% of the total Ig-seq dataset (2,000 sequences) in 570k CPU hours. Finally, the paired reads with CDR-H3 sequences longer than 16 amino acids were not included in the structural analysis as the modeling accuracy of such loops is currently low. This emphasizes the challenges of modeling longer CDR-H3 configurations (94, 96). Hence, novel fast and reliable CDR-H3 *ab initio* prediction as well as technologically optimized paired VH/VL gene Ig-seq are urgently needed for improved Ig-seq data modeling and interrogation.

RosettaAntibody (98) is a well-established antibody modeling tool and is able to structurally model sequence data; however, its run times make it difficult to structurally characterize the millions of sequences that are gathered during a typical Ig-seq experiment. For this reason, streamlined approaches are being developed to tackle the structural annotation of Ig-seq datasets. For instance, Nowak et al. (44) performed the structural clustering analysis of CDR-L3 of two large Ig-seq datasets: 200k paired Ig-seq sample from Dekosky et al. (15) and 9-m in-house UCB Pharma Ltd sequences as well as a database of 71k antibody sequences [DIGIT (111)]. Every CDR-L3 sequence was submitted to HMMER (112) to assign it to a length-independent cluster. This is the first instance of structurally mapping the entirety of an Ig-seq dataset. The method can be extrapolated to any non-H3 CDR to provide structural annotation of sampling of loop shapes as well as to identify yet uncharacterized loop configurations.

Structural characterization of large sequence sets can be extended to the entire Fv region. The modeling method, ABodyBuilder, was used to predict structures of 6,000 paired antibody sequences from public repositories (8). The average modeling time per 1,000 antibody sequences was 567 CPU hours compared with 285,000 CPU hours using RossettaAntibody (45). ABodyBuilder produces model accuracies that are in line with the AMA-II values (80). Using tools such as ABodyBuilder, one can perform large-scale structural modeling of Ig-seq data. Such structural characterization of Ig-seq similarity/difference would allow more accurate inter-molecule comparisons and assessment of developability. The structural software outlined in this manuscript together with other tools that are often employed in computational/structural annotation of antibody sequences is summarized in Table 1.

**Table 1:** Summary of currently available resources for computational/structural annotation of antibody sequences.

Tool type	Tool name and reference	Short tool description
ANTIBODY NUMBERING	ANARCI (113)	Variety of schemes (North, Chothia, Kabat, IMGT, AHo). Both online and command line versions are available
ANTIBODY NUMBERING	Abnum (71)	Online numbering tool that operates with Kabat and Chothia schemes
SEQUENCE ANALYSIS	IgBLAST (114)	Nucleotide and amino-acid antibody sequence analysis in IMGT and KABAT schemes
SEQUENCE ANALYSIS	IMGT/HighV-QUEST (115)	Online antibody nucleotide sequence analysis in IMGT numbering scheme
STRUCTURE DATABASE	SabDab (29)	Weekly updating database of all publically available antibody structures.
STRUCTURE/SEQUENCE DATABASE	abYsis (116)	Database of antibody structures and sequences
SEQUENCE DATABASE	DIGIT (111)	Database of antibody sequences
ANTIBODY MODELING	ABodyBuilder (8)	Homology modeling (30 s per model)
ANTIBODY MODELING	PIGSPro (86)	Homology modeling
ANTIBODY MODELING	Kotai Antibody Builder (95)	Homology modeling (90 min per model)
ANTIBODY MODELING	Accelrys (101)	Hybrid modeling (30 min per model)
ANTIBODY MODELING	RosettaAntibody (87)	*Ab initio* modeling (482 CPU hours per model)
ANTIBODY MODELING (COMMERCIAL)	Chemical Computing group (80)	Homology modeling tool combined with molecular dynamics (30 min per model)
CDR-H3 MODELING	Sphinx (102)	Length-independent hybrid modeling (30 min per model)
CDR-H3 MODELING	PLOP (99)	*Ab initio* modeling
CDR-H3 MODELING	FREAD (85)	Homology modeling (2 min per model)
PARATOPE PREDICTION	Paratome (117)	Structural consensus to identify additional antigen recognizing regions outside the CDRs
PARATOPE PREDICTION	i-Patch (118)	Statistical inference to devise a likelihood for a position to form a potential contact
PARATOPE PREDICTION	proABC (119)	Sequence-based method that leverages machine learning to predict residues that form interactions

*Many of these tools have online presence and links to these are available on our website http://antibodystructure.org*.

## Conclusion

The ability to engineer better antibody-based therapeutics relies on our knowledge of the exact sequence and the 3D shape of individual molecules within the antibody repertoire. Next-generation sequencing methodologies that can yield millions of immunoglobulin gene sequences in a single sequencing run have already given insights into the steady-state and antigen-stimulated B-cell receptor repertoire (12, 32). On the other hand, low-throughput techniques such as X-ray crystallography can provide detailed information about individual antibody structures. Computational methodologies can offer a bridge between the two fields by allowing structural annotation of Ig-seq experiments (8, 44, 45). Availability of antibody structures and maturity of modeling techniques means it is now possible to perform large-scale structural characterizations of Ig-seq samples. This enriched structural content can be used to perform more precise characterization of antibodies allowing inter-antibody comparisons and grouping of structurally similar sequences (that may not be possible on the sequence level) as well as annotation of developability information. Large-scale Ig-seq datasets can also direct computational tools for targeted interrogation of antibody structural space. Statistical knowledge of the distribution of the antibody structures and sequences can offer crystallographers an idea of the common but currently unknown antibody variants. The Ig-seq and structural communities will benefit from cross-fertilization of ideas and methodologies. Together they will advance our knowledge of the antibodies in health and disease and pave the way for more advanced antibody-based therapeutics.

## Author Contributions

All authors contributed to the development of writing of the manuscript.

## Conflict of Interest Statement

The authors declare that the research was conducted in the absence of any commercial or financial relationships that could be construed as a potential conflict of interest.
